# Molecular mechanisms and treatment responses of pulmonary fibrosis in severe COVID-19

**DOI:** 10.1186/s12931-023-02496-1

**Published:** 2023-08-09

**Authors:** Emma J. Kooistra, Kilian Dahm, Antonius E. van Herwaarden, Jelle Gerretsen, Melanie Nuesch Germano, Karoline Mauer, Ruben L. Smeets, Sjef van der Velde, Maarten J. W. van den Berg, Johannes G. van der Hoeven, Anna C. Aschenbrenner, Joachim L. Schultze, Thomas Ulas, Matthijs Kox, Peter Pickkers

**Affiliations:** 1 Department of Intensive Care Medicine, Radboud University Medical Center, Postbus 9101, 6500 HB Nijmegen, The Netherlands; 2 Radboud Center for Infectious Diseases, Radboud University Medical Center, 6500 HB Nijmegen, The Netherlands; 3 Translational Pediatrics, Department of Pediatrics, University Hospital Wuerzburg, 97080 Würzburg, Bavaria Germany; 4 PRECISE Platform for Single Cell Genomics and Epigenomics, German Center for Neurodegenerative Diseases, University of Bonn, Bonn, Germany; 5 Radboudumc Laboratory for Diagnostics, Department of Laboratory Medicine, Radboud University Medical Center, 6500 HB Nijmegen, The Netherlands; 6 Life and Medical Sciences (LIMES) Institute, University of Bonn, Bonn, Germany; 7 Laboratory for Medical Immunology, Department of Laboratory Medicine, Radboud University Medical Center, 6500 HB Nijmegen, The Netherlands; 8 Systems Medicine, Deutsches Zentrum für Neurodegenerative Erkrankungen (DZNE), Bonn, Germany

**Keywords:** Acute respiratory distress syndrome, Coronavirus disease 2019, Dexamethasone, Pulmonary fibrosis, Transcriptomics

## Abstract

**Background:**

Coronavirus disease 2019 (COVID-19) patients can develop pulmonary fibrosis (PF), which is associated with impaired outcome. We assessed specific leukocytic transcriptome profiles associated with PF and the influence of early dexamethasone (DEXA) treatment on the clinical course of PF in critically ill COVID-19 patients.

**Methods:**

We performed a pre-post design study in 191 COVID-19 patients admitted to the Intensive Care Unit (ICU) spanning two treatment cohorts: the *pre-DEXA*- (n = 67) and the *DEXA-cohort* (n = 124). PF was identified based on radiological findings, worsening of ventilatory parameters and elevated circulating PIIINP levels. Longitudinal transcriptome profiles of 52 *pre-DEXA* patients were determined using RNA sequencing. Effects of prednisone treatment on clinical fibrosis parameters and outcomes were analyzed between PF- and no-PF-patients within both cohorts.

**Results:**

Transcriptome analyses revealed upregulation of inflammatory, coagulation and neutrophil extracellular trap-related pathways in PF-patients compared to no-PF patients. Key genes involved included *PADI4*, *PDE4D*, *MMP8*, *CRISP3*, and *BCL2L15*. Enrichment of several identified pathways was associated with impaired survival in a external cohort of patients with idiopathic pulmonary fibrosis. Following prednisone treatment, PF-related profiles reverted towards those observed in the no-PF-group. Likewise, PIIINP levels decreased significantly following prednisone treatment. PF incidence was 28% and 25% in the pre-DEXA- and DEXA-cohort, respectively (p = 0.61). ICU length-of-stay (*pre-DEXA*: 42 [29–49] vs. 18 [13–27] days, p < 0.001; *DEXA*: 42 [28–57] vs. 13 [7–24] days, p < 0.001) and mortality (pre-DEXA: 47% vs. 15%, p = 0.009; DEXA: 61% vs. 19%, p < 0.001) were higher in the PF-groups compared to the no-PF-groups within both cohorts. Early dexamethasone therapy did not influence these outcomes.

**Conclusions:**

ICU patients with COVID-19 who develop PF exhibit upregulated coagulation, inflammation, and neutrophil extracellular trap-related pathways as well as prolonged ICU length-of-stay and mortality. This study indicates that early dexamethasone treatment neither influences the incidence or clinical course of PF, nor clinical outcomes.

**Supplementary Information:**

The online version contains supplementary material available at 10.1186/s12931-023-02496-1.

## Background

Patients with Coronavirus Disease 2019 (COVID-19)-induced Acute Respiratory Distress Syndrome (ARDS) are at risk of subsequent complications such as a pathological fibroproliferative response [1, 2]. Pulmonary fibrosis (PF) is associated with challenges in mechanical ventilation, prolonged length of stay (LOS) in ICU, higher mortality rates, and chronic symptoms in survivors [3–7].

It is challenging to detect PF at an early stage of ARDS. High N-terminal pro-peptide of type III procollagen (PIIINP) levels in bronchoalveolar lavage (BAL) fluid [8], as well as PIIINP in blood may be used. Also, other circulating fibrosis biomarkers such as hepatocyte growth factor (HGF) [9] and Macrophage Inflammatory Protein-3 alpha (MIP-3α) [10] could be of value. The mechanisms underlying the development of PF are largely unexplored, while knowledge of these pathways may aid early diagnosis and novel treatment targets. Currently, PF in patients with non-COVID-19 ARDS is treated with corticosteroids, which is effective in reducing time on mechanical ventilation and ICU-LOS, especially in those with elevated biomarker concentrations [11, 12].

Initially, care for critically ill COVID-19 patients was limited to supportive treatment. However, since early treatment with the corticosteroid dexamethasone (DEXA) was shown to be beneficial [13], hospitalized COVID-19 patients requiring oxygen suppletion were all treated with DEXA. It is however unknown whether DEXA treatment influences the incidence or severity of PF and whether or not it affects the therapeutic efficacy of later corticosteroid treatment in patients who develop PF. The primary aim of this study in critically ill COVID-19 patients was therefore twofold: (1) to explore transcriptome profiles associated with PF and the response to treatment using longitudinal RNA sequencing of circulating leukocytes. (2) to determine the influence of early dexamethasone treatment on the incidence and time to development of PF, and to assess the therapeutic efficacy of steroids to treat PF both before and after the introduction of early dexamethasone as standard care for critically ill COVID-19 patients.

## Methods

### Study design and participants

In this prospectively designed pre-post design cohort study, all adult COVID-19 patients admitted to the ICU of Radboud University Medical Center (Radboudumc, Nijmegen, The Netherlands) between March 2020 and April 2021 were screened for inclusion. Patients with comorbidities that might significantly influence the disease course and clinical outcomes (e.g. immunocompromised patients) were excluded. This study was carried out in accordance with the applicable rules concerning the review of research ethics committees and informed consent in the Netherlands. All patients or legal representatives were informed about the details of this cohort study and could decline to participate.

Included patients were divided into two cohorts: patients who were not treated with DEXA (*pre-DEXA*-*cohort,* March 2020–August 2020) and patients who received DEXA (6 mg/day, intravenously for 10 days) as part of standard COVID-19 care in accordance to the RECOVERY criteria [13] (*DEXA-cohort*, August 2020–April, 2021). A subgroup of the *DEXA-cohort* was also treated with the interleukin (IL)-6 receptor antagonist tocilizumab as part of standard COVID-19 care (single dose of 8 mg/kg, intravenously) [14]. Details on the sensitivity analyses performed in this subgroup are provided in the Additional file 1. Both cohorts were subdivided into groups of patients who were assessed to have developed PF while still in ICU and were treated with prednisone (start dose of 1 mg/kg twice daily, intravenously, *PF-groups*) and groups of patients who were not (*no-PF-groups*). In the absence of validated diagnostic criteria of PF, the diagnosis, and therefore the indication for prednisone treatment was at the discretion of the treatment team. All patients were discussed daily in a multidisciplinary meeting including over 15 medical experts, suspicion of PF and initiation of prednisone treatment was based on a combination of radiological findings, worsening ventilatory parameters (e.g. lower PaO_2_/FiO_2_ ratio, lower lung compliance and increased ventilatory ratio as a measure of impaired ventilation and increase in dead space ventilation), and an increase in PIIINP plasma levels that were measured three times per week. To analyze the kinetics of fibrosis biomarkers in the days prior to and following the day on which prednisone treatment for PF was initiated, serial data were aligned on the first day of prednisone treatment for PF (PF-day 0). For patients of the no-PF-groups, data were aligned on the median start day of late prednisone treatment in both cohorts separately to correct for time-dependent effects in this group [15, 16].

### RNA sequencing

To explore underlying molecular mechanisms of PF development and treatments responses, we performed RNA sequencing on leukocytes isolated from a total of 52 PF- and no-PF-patients of the *pre-DEXA-*cohort. We used co-expression network analysis on these longitudinal RNA sequencing data using our established hCocena pipeline [17] to identify similarly regulated genes across samples and group these genes into modules. We applied this approach to samples obtained up to day 0 (when prednisone treatment was initiated in PF patients), to identify genes associated with the development of PF (pre-alignment day analysis). To assess the transcriptome response to treatment of PF with prednisone, we applied the same analysis pipeline to samples obtained from day 0 onwards (post-alignment day analysis). See Additional file 1 for a detailed description of RNA sequencing and analysis procedures.

### Clinical data and biomarker measurements

See Additional file 1.

### Statistical analysis

Differences in baseline characteristics and clinical outcomes between the PF- and no-PF-groups were analyzed using Mann–Whitney U and Fisher’s exact tests for continuous and categorical data, respectively. Differences in kinetics of serially measured data were analyzed using linear mixed effect model analysis on log-transformed data followed by post-hoc Sidak’s multiple comparisons tests. ICU-LOS and mortality were analyzed using log-rank tests during 60 days following ICU admission. A more detailed description of the statistical analysis is presented in Additional file 1.

## Results

### Patient characteristics

**Fig. 1:**
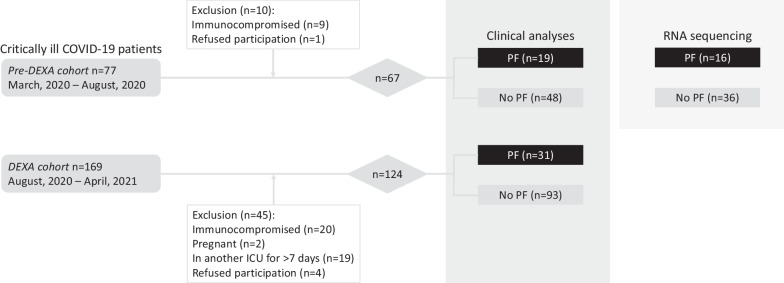
Patient flowchart. *COVID-19* Coronavirus Disease 2019, *DEXA* dexamethasone, *ICU* intensive care unit, *PF* pulmonary fibroproliferation

The *pre-DEXA-cohort* and the *DEXA*-*cohort* consisted of 67 and 124 patients, respectively (Fig. 1). Baseline characteristics of both cohorts are listed in Table 1. Prednisone treatment for PF was initiated on day 16 [12–21] and day 19 [14–23] following ICU admission in the *pre-DEXA*- and *DEXA-cohorts*, respectively (p = 0.11, Table 1). No relevant baseline demographic differences were present between the PF- and no-PF-groups within both cohorts. Furthermore, within the *DEXA-cohort*, no difference in the proportion of patients who were also treated with tocilizumab as standard COVID-19 care was present between the PF- and no-PF-groups (65% vs. 60%, p = 0.83, Table 1). Also, when comparing the PF-groups between both cohorts, no significant differences were present in patient characteristics and PF-free days from hospital admission onwards (Table 1).

**Table 1:** Patient characteristics of the *pre-DEXA-* and *DEXA-cohort*s

	*Pre-DEXA-cohort* (n = 67)	*DEXA-cohort* (n = 124)	p-value
Age, years	65 [58–72]	65 [56–72]	0.89
Sex, male	50 (75)	84 (68)	0.41
BMI, kg/m^2^	27.7 [24.9–30.8]	29.4 [26.2–33.3]	0.005
APACHE II	15 [12–19]	16 [13–20]	0.08
Days first COVID-19 signs until ICU admission	11 [7–13]	10 [7–12]	0.46
Medical history			
Renal insufficiency	1 (1)	3 (2)	1.00
Metastatic neoplasm	5 (7)	2 (2)	0.053
Immunological insufficiency	1 (1)	6 (5)	0.43
COPD	6 (9)	13 (10)	0.81
Diabetes mellitus	15 (22)	32 (26)	0.73
Hypertension	33 (49)	63 (51)	0.88

Data presented as n (%) or median with interquartile ranges ([IQR]). P-values were calculated using Mann–Whitney U and two-sided Fisher’s exact tests for continuous and categorical data, respectively

*DEXA* dexamethasone, *PF* pulmonary fibrosis, *BMI* body mass index, *COVID-19* corona virus disease 2019, *ICU* intensive care unit, *COPD* chronic obstructive pulmonary disease

### Transcriptome analysis

**Fig. 2:**
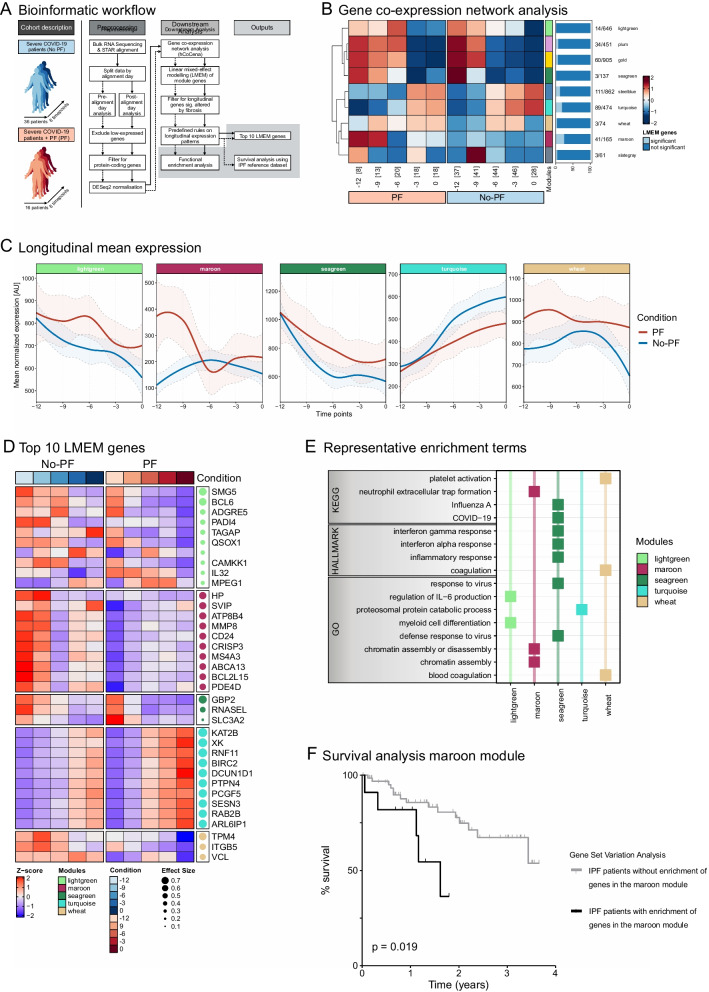
Summary of bulk RNA-seq data pre-alignment day (day 0, the day prednisone treatment was initiated in patients with PF). **a** Depicts an overview of the complete cohort and general workflow per data set (dashed: pre-alignment day data set; solid: post-alignment day data set). **b** Shows the expression profile across the time points prior to the alignment day per module in a heatmap split by condition. The amount of samples per timepoint is displayed in brackets. Tiles are colored based on the group fold change (GFC) and modules are represented by their respective colors. Percentage of LMEM genes per module are depicted in a barplot (colored based on significance in the LMEM) and total numbers are shown as a ratio of LMEM genes to module genes. **c** Displays the mean expression per fibrosis-related module filtered by the LMEM genes over time prior to the alignment day. Lines and confidence intervals are colored according to the condition. **d** Depicts the mean expression of the top 10 LMEM genes per fibrosis-related module ordered by effect size for all conditions and time points prior to the alignment day. Modules are colored accordingly and effect size is indicated by the dot size. **e** Shows significant representative functional enrichment terms from GO and KEGG database as well as the hallmark gene set of the Molecular Signature Database per fibrosis-related module. Modules names are displayed on the x-axis and the respectively colored squares indicate the enrichment of a functional term in the module. **f** Displays the Kaplan–Meier plot of patients with idiopathic pulmonary fibrosis with and without enrichment of genes in the maroon module. Lines are colored based on the enrichment of the LMEM genes in the maroon module in the reference dataset using GSVA

In blood samples obtained up to day 0 (when prednisone treatment was initiated in PF patients), we identified nine co-expressed modules associated with the development of PF across a total of 3775 genes included in the analysis (pre-alignment day analysis, Fig. 2a, b). These modules are designated by colors gold to wheat (Fig. 2b). Based on linear regression analysis and a predefined set of rules (see Fig. 2a and Additional file 1), we focused on five modules associated with PF: seagreen, lightgreen, maroon, and wheat (upregulated in PF-patients) and turquoise (downregulated in PF-patients). Differential expressed genes over time were visualized by wave plots (Fig. 2c, wave plots of all modules provided in Additional file 1: Fig. S1) and heatmaps of the top 10 significant genes ranked by effect size (Fig. 2d, genes of all modules provided in Additional file 1: Fig. S1). Functional Enrichment Analysis (FEA) on these modules identified associated gene signatures with distinct functional characteristics related to fibrosis (Fig. 2e, all associated gene signatures provided in Additional file 1: Fig. S1). For instance, ‘*inflammatory response*’, ‘*interferon (IFN)-γ response*’, ‘*IFN-α responses*’, ‘*response to virus*’, ‘*COVID-19*’ and ‘*influenza*’ are enriched in the seagreen module, in keeping with the fact that inflammation is an important driver of fibrotic processes [18]. Hence, these data suggest a more pronounced response to (viral) infections, leading to more severe inflammation in COVID-19 patients who developed PF compared to COVID-19 patients who did not. In accordance, ‘*regulation of interleukin-6 production*’ and ‘*myeloid cell differentiation*’ were enriched in the lightgreen module and play key roles in both inflammation and development of PF [19–21]. The wheat module showed enrichment for ‘*coagulation*’ and ‘*platelet activation*’, and previous work has shown that the coagulation pathway is involved in fibroproliferative responses [22]. Accordingly, prevalence rates of pulmonary embolisms (PE) during stay in ICU and use of therapeutic low molecular weight heparins (LMWH) were compared between PF and no-PF groups. No differences in prevalence rates of PE were present between PF and no-PF groups (71% vs. 65%, respectively, p = 1.00). Therapeutic dosage of LMWH were administered in 75% of PF patients compared to 42% of no-PF patients (p = 0.04). ‘*Neutrophil extracellular trap (NET) formation*’ and ‘*chromatin assembly*’, were enriched in the maroon module. Interestingly, the release of NETs has been shown to play a role in the development of organ fibrosis [23] and their release is dependent on histone modification by peptidylarginine deiminase 4 (PADI4) [23, 24], which was one of the top 10 genes in the lightgreen module (Fig. 2d). The turquoise module which was downregulated in PF-patients, showed enrichment of ‘*proteasomal protein catabolic process*’ and ‘*ubiquitin-mediated proteolysis*’. Dysregulation of the ubiquitin–proteasome pathway is linked to multiple conditions, including fibrotic diseases [25], implicating that the ubiquitin–proteasome pathway is less functional in COVID-19 patients with PF. Finally, several specific genes which were distinctly upregulated in PF patients have previously been linked to fibrotic processes, including *PDE4D* [26], *MMP8* [27], *CRISP3* [28], and *BCL2L15* [29] (all in maroon module, Fig. 2d). Additionally, to explore relationships between the gene modules and clinical outcomes of fibrosis, we performed gene set variation analysis on leukocyte gene expression data of a published cohort of patients with idiopathic pulmonary fibrosis (IPF, Fig. 2a) [30]. Four-year survival of IPF patients who showed enrichment of the genes in each module was compared to outcome of patients who exhibited no enrichment. Strikingly, survival of IPF patients who showed enrichment for genes in the maroon module was significantly worse (p = 0.019, Fig. 2f, survival plots for all modules provided in Additional file 1: Fig. S2).

**Fig. 3:**
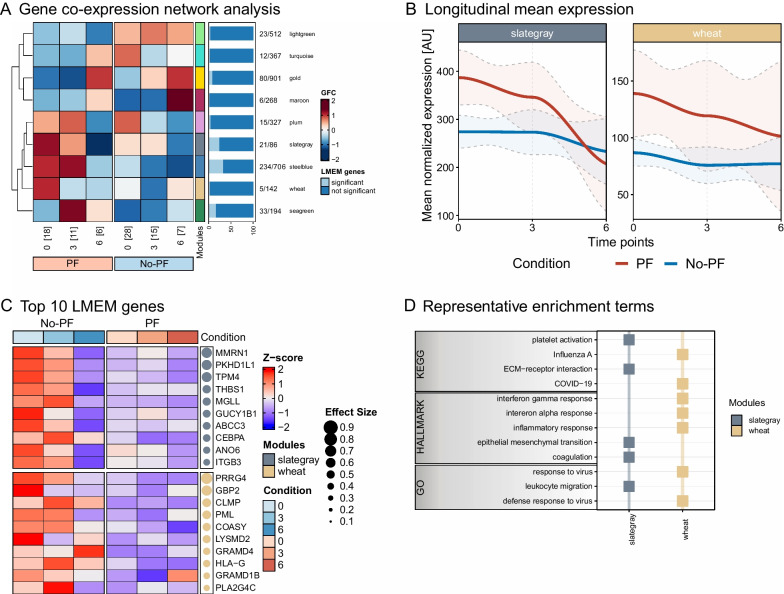
Summary of bulk RNA-seq data post-alignment day (day 0, the day prednisone treatment was initiated in patients with PF). **a** Shows the expression profile across the time points post-alignment day per module in a heatmap split by condition. The amount of samples per timepoint is displayed in brackets. Tiles are colored based on the group fold change (GFC) and modules are represented by their respective colors. Percentage of LMEM genes per module are depicted in a barplot (colored based on significance in the LMEM) and total numbers are shown as a ratio of LMEM genes to module genes. **b** Displays the mean expression per fibrosis-related module filtered by the LMEM genes over time post-alignment day. Lines and confidence intervals are colored according to the condition. **c** Depicts the mean expression of the top 10 LMEM genes per fibrosis-related module ordered by effect size for all conditions and time points post-alignment day. Modules are colored accordingly and effect size is indicated by the dot size. **d** Shows significant representative functional enrichment terms from GO and KEGG database as well as the hallmark gene set of the Molecular Signature Database per fibrosis-related module. Modules names are displayed on the x-axis and the respectively colored squares indicate the enrichment of a functional term in the module

The transcriptome response to treatment of PF with prednisone was assessed in blood samples obtained from day 0 onwards (post-alignment day analysis, see Fig. 2a). This analysis revealed nine co-expression modules (Fig. 3a). Applying the linear regression analysis and the predefined set of rules, led us to focus on two modules: slategray and wheat (Fig. 3b, c, wave plots and top 10 genes of all modules are provided in Additional file 1: Fig. S3). Genes in both modules were upregulated in the PF-group on day 0 and converged towards the no-PF-group afterwards, suggesting a treatment effect. Similar to the pre-alignment day analysis, both modules are enriched for multiple inflammatory and coagulation pathways (Fig. 3d, all associated gene signatures provided in Additional file 1: Fig. S3). Furthermore, the slategray module showed enrichment for ‘*epithelial mesenchymal transition*’, which was previously implicated in the development of organ fibrosis [31–33].

There was no evidence that any of the module genes were regulated differentially between PF and no-PF groups by pro-fibrotic cytokines e.g. TGF-β.

### Fibrosis biomarkers and ventilatory parameters

**Fig. 4:**
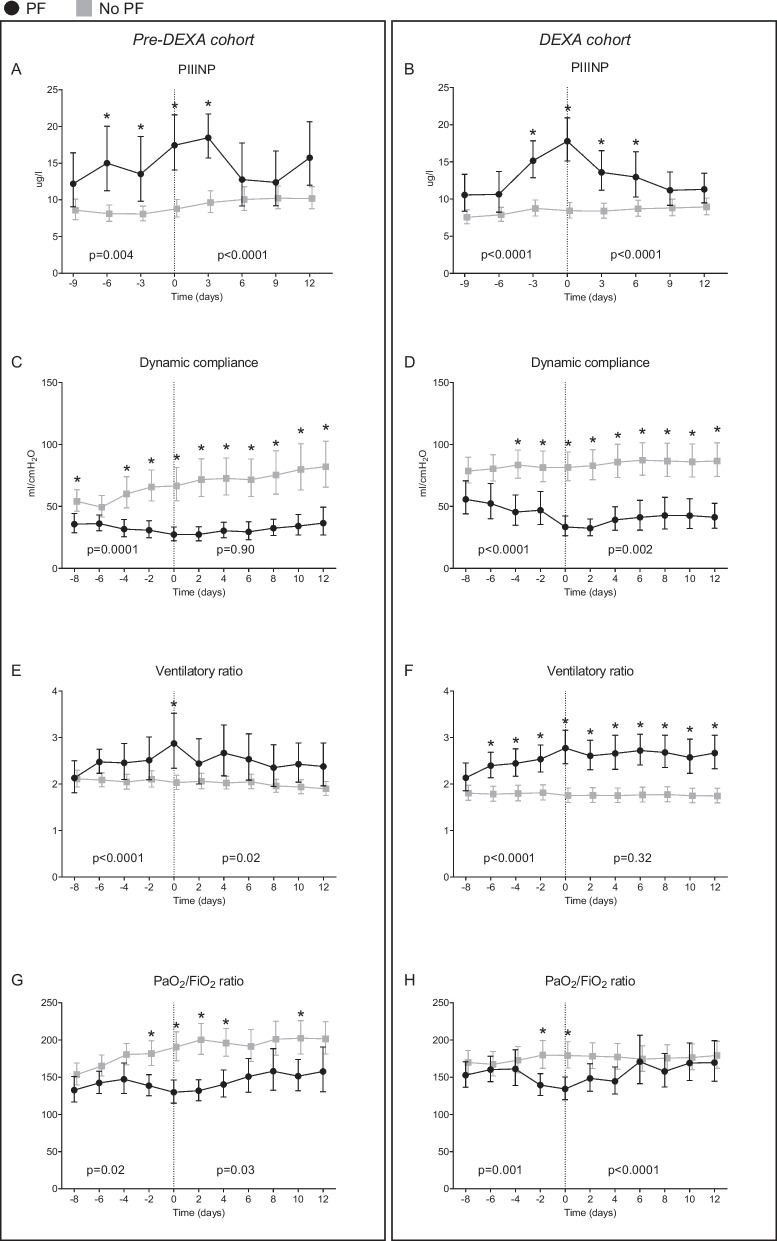
Circulating procollagen type III concentrations and clinical parameters. Differences between the pulmonary fibrosis (PF)- and no-PF-groups in kinetics of circulating procollagen type III (PIIINP) in **a** *pre-DEXA-cohort* and **b** *DEXA-cohor*t, dynamic lung compliance in **c** *pre-DEXA-cohort* and **d** *DEXA-cohort,* ventilatory ratio in **e** *pre-DEXA-cohort* and **f** *DEXA-cohort*, and PaO_2_/FiO_2_ ratio in **e** *pre-DEXA-cohort* and **f** *DEXA-cohort* within 9 days (PIIINP) or 8 days (ventilatory parameters) prior to and 12 days following the alignment day (PF-day 0, start of prednisone treatment in the PF-groups). P-values on the left and the right of each panel reflect between-group differences over time for the days prior to and following PF-day 0, respectively, and were calculated using linear mixed models analysis (time * group interaction factor). Data presented as geometric mean with 95% confidence intervals. *p-value < 0.05 on the corresponding timepoint, calculated using Sidak’s post-hoc multiple comparisons tests

Peak PIIINP levels in the PF-groups were observed on day 3 following start of prednisone treatment and on the day prednisone treatment was initiated for PF (day 0) for the *pre-DEXA*- and *DEXA-cohorts*, respectively. PIIINP levels decreased significantly following prednisone treatment (Fig. 4ab). These kinetics were not observed in the no-PF-groups (Fig. 4ab). Unlike PIIINP, no significant between-group differences in circulating levels of HGF and MIP-3α within both cohorts were present (Additional file 1: Fig. S4). Following initiation of prednisone treatment, the dynamic lung compliance remained lower in the PF-groups of both cohorts compared with the no-PF-groups during the entire follow-up period (Fig. 4c, d). In the *pre-DEXA-cohort*, the ventilatory ratio decreased following initiation of prednisone treatment in the PF-group, whereas no relevant changes in ventilatory ratio were observed in the no-PF-group (Fig. 4e). In the *DEXA-cohort*, the ventilatory ratio of the PF-group remained higher compared to the no-PF-group on all ensuing timepoints following initiation of prednisone treatment (Fig. 4f). In the *pre-DEXA-*cohort, PaO_2_/FiO_2_ ratio of the PF-group remained lower compared to the no-PF-group for several days following start of prednisone treatment, while this was not the case in the *DEXA-cohort* (Fig. 4g, h). Kinetics and values on individual timepoints of all ventilatory parameters were similar between PF patients of both cohorts (Additional file 1: Fig. S5). So, overall, early DEXA treatment did not influence the subsequent response to steroid therapy in PF patients.

### Clinical outcomes

**Fig. 5:**
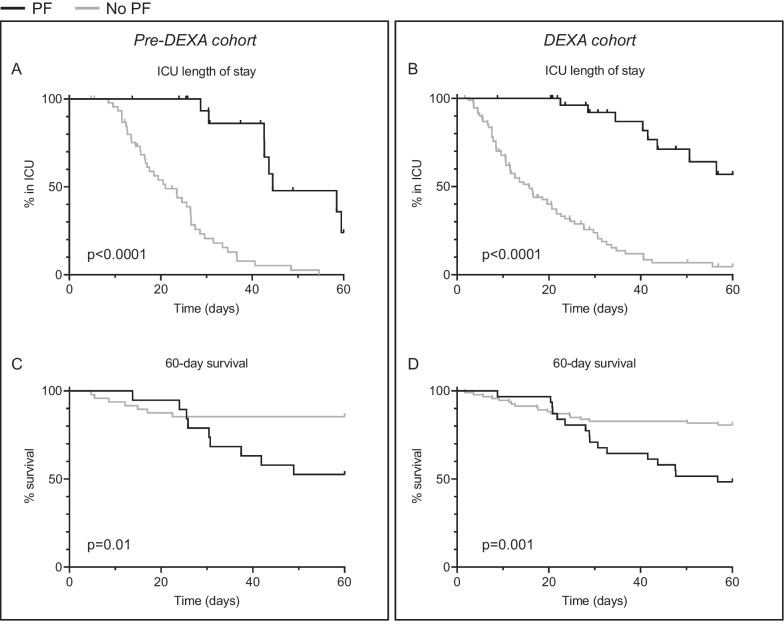
Clinical outcomes. Differences between the pulmonary fibrosis (PF)- and no PF-groups in length of stay (LOS) in the intensive care unit (ICU) in **a** the *pre-DEXA-cohort* and **b** the *DEXA-cohort*, and 60-day hospital mortality in **c** the *pre-DEXA-cohort* and **d** the *DEXA-cohort*. Kaplan–Meier curves are depicted and p-values were calculated using log-rank tests. For analysis of 60-day hospital mortality, patients who were discharged alive from the hospital or were still in the ICU or hospital on day 60 were censored at day 60. Numbers at risk are shown below graphs

PF incidence was 28% and 25% in the pre-DEXA- and DEXA-cohorts, respectively (p = 0.61). Time on ventilator, LOS in ICU and mortality were higher in the PF-groups compared to the no-PF-groups within both cohorts (Table 1, Fig. 5). Furthermore, within both cohorts, PF-patients who survived their ICU stay had a prolonged time on mechanical ventilation and ICU stay compared to no-PF-patients who survived. None of the clinical outcomes differed between the PF-groups of both cohorts (Table 1), again indicating no influence of early DEXA treatment on these clinical response to steroid treatment for PF. When dividing the *DEXA*-*cohort* into subgroups of patients who were also treated with tocilizumab and those who were not, similar differences in clinical outcomes between the PF- and no-PF-groups were observed as in the main analysis (Additional file 1: Table S1). Furthermore, no differences in clinical outcomes were present between PF-patients who were co-treated with tocilizumab and PF-patients who were not (Additional file 1: Table S1).

## Discussion

Our study reveals that several genes and signaling pathways that were previously linked to organ fibrosis are upregulated in critically ill COVID-19 patients who develop PF, including inflammatory processes, coagulation, and NET-related pathways. Furthermore, we demonstrate that some of the identified pathways are associated with worse long-term outcomes of fibrotic diseases. Following initiation of steroid treatment for PF, multiple upregulated pathways in the PF-group converged towards expression levels observed in the no-PF-group. Likewise, circulating PIIINP levels reverted to concentrations similar to those observed in the no-PF-group following treatment of PF with steroids. Whereas several clinical ventilatory parameters also stabilized or improved after treatment, these largely remained worse in PF-patients compared to no-PF-patients. Importantly, this treatment response was not influenced by early dexamethasone treatment. Finally, PF was associated with a prolonged length of stay in the ICU and higher mortality rates, which was also not influenced by early dexamethasone treatment or co-treatment with tocilizumab.

Up to now, multiple studies have described long-term symptoms of COVID-19 in both ICU and non-ICU COVID-19 patients, including pulmonary sequalae [34–37]. The exact underlying mechanisms for these long-lasting symptoms are still unclear, but the development of PF likely plays a role. Therefore, and because of its high mortality, it is of paramount importance to detect and treat PF at an early stage. In non-COVID-19 ARDS patients, it was shown that corticosteroid treatment is effective in shortening ICU-LOS and reducing mortality rates [11]. Accordingly, we hypothesized that early dexamethasone treatment would result in a lower incidence or less severe course of excessive PF and subsequent more pronounced improvement of clinical pulmonary outcomes in patients of our *DEXA-cohort* compared to the *pre-DEXA*-cohort. In contrast, we did not observe differences in incidence rates or clinical outcomes between both cohorts.

Although it did not reach statistical significance, time from ICU admission until the initiation of prednisone to treat PF was 3 days later in the *DEXA-cohort*. One may argue that prolongation of the early dexamethasone treatment as standard care in this subgroup of critically ill COVID-19 patients could further delay or even prevent the development of PF. Additionally, the dose of dexamethasone used as standard treatment for COVID-19 is considerably lower than the equivalent corticosteroid dose of prednisone used for the treatment of PF. For example, a patient with PF of 80 kg would be treated with 160 mg prednisone daily, approximately equivalent to 24 mg dexamethasone [38], and thus several times higher than the 6 mg dexamethasone dose used as standard treatment for COVID-19. Therefore, we cannot exclude that prolongation of treatment, or increasing the dosage of early dexamethasone may mitigate the development of PF and improve clinical outcomes in critically ill COVID-19 patients. Of interest, a recently published study compared the effects of daily administration of 6 mg and 12 mg dexamethasone for 10 days in 982 severely ill COVID-19 patients and showed better clinical outcomes in the 12 mg group, while the incidence of serious adverse effects was similar [39]. To investigate the effects of early dexamethasone treatment on incidence rates and mortality of all hospitalized COVID-19 patients and the effects of prolongation/intensification of dexamethasone treatment on the development of PF in critically ill COVID-19 patients, randomized controlled trials should be performed.

Our longitudinal transcriptome analysis provided clues for novel therapeutic targets for prevention or treatment of PF in critically ill COVID-19 patients. One of the most strongly upregulated genes in PF-patients, *MMP8*, is related to bleomycin-induced fibrosis in mice [27, 40], that treatment with MMP8 inhibitors may be beneficial. Similar, inhibition of PDE4, also markedly upregulated in PF patients, prevented PF in bleomycin-treated mice [26]. Interestingly, the PDE4 inhibitor roflumilast is already licensed for the treatment of severe COPD and asthma [41, 42].

This study has several limitations. First, early dexamethasone treatment was not randomized. As a consequence, bias related to the initial response to dexamethasone is likely present, especially because this treatment is often started on the ward. Therefore, it is possible that several patients of the *pre-DEXA-*cohort would not have required ICU admission if they would have received dexamethasone on the ward. On the other hand, our data are as observed in current clinical practice. Second, later on, patients were treated with prednisone when PF was identified based on radiological findings, worsening of ventilatory parameters and elevated circulating PIIINP levels which were available to the treating physicians. Ideally, PF should be diagnosed based on high PIIINP levels in BAL fluids and typical high-resolution computed tomography (HRCT) images [4, 8]. However, during the COVID-19 pandemic, it was not feasible to perform repeated BALs and HRCTs, and circulating PIIINP levels have shown promise for evaluation of disease progression and treatment efficacy in non-COVID-19 ARDS patients [12, 43]. Also, the differences in transcriptome profiles between PF- and no-PF-patients support the PF diagnosis, and the changes in PIIINP kinetics, pulmonary parameters, and gene expression patterns following prednisone treatment illustrate therapeutic efficacy. Third, the transcriptome analyses were performed on total leukocytes. With this approach, changes in blood count differential (i.e. the percentages of e.g. monocytes, lymphocytes, and neutrophils) influence gene expression levels. Unfortunately, we do not have data available on blood count differentials to assess the magnitude of this effect in our analyses. Fourth, since the relatively small sample size, our study was possibly underpowered to draw conclusions on the effects of early dexamethasone treatment on incidence and clinical outcomes of COVID-19 patients with PF. However, we did not find any (non-significant) indications to a major influence of early dexamethasone treatment on these outcomes. Last, the observational design of our study could have introduced confounding because of the rapidly increasing knowledge of the disease and the so-called *learning curve* during the pandemic. Since we compared data of two cohorts of COVID-19 patients who were admitted to the ICU during different periods in time, the possibility that the introduction of dexamethasone and tocilizumab as standard treatment in COVID-19 was not the only difference in treatment between both cohorts has to be acknowledged. For example, differences in virulence of the dominant SARS-CoV-2 strain during each period of time might also be of influence on our study outcomes. Preferably, a RCT in hospitalized COVID-19 patients should be performed to more accurately determine the effects of early dexamethasone treatment on the incidence, clinical course and outcomes of PF, although this would now raise ethical dilemmas.

## Conclusions

In critically ill COVID-19 patients who develop PF, coagulation, inflammation and NET-related pathways are upregulated, ICU-LOS is prolonged and mortality is higher. This study indicates that early dexamethasone treatment neither influences the incidence or clinical course of PF, nor the outcomes of this subgroup of critically ill COVID-19 patients. Steroid treatment normalized PF-related RNA profiles and PIIINP levels, while clinical parameters stabilized, but remained aberrant compared to critically ill COVID-19 patients without signs of PF.

### Supplementary Information

**Additional file 1.** Additional Methods, Additional Table S1 and Additional Figures S1–S5.

## Data Availability

The RNA sequencing data are made available at the European Genome-Phenome Archive (EGA) under accession numbers EGAS00001005735 and EGAS00001006407, which is hosted by the EBI and the CRG. The R package to bin and align longitudinal clinical data is available here: 10.5281/zenodo.6594554. Other datasets used and/or analysed during the current study are available from the corresponding author on reasonable request.
