# Supplementary material for: Molecular mechanisms and treatment responses of pulmonary fibrosis in severe COVID-19
Source: Respir Res. 2023 Aug 9;24:196. doi: 10.1186/s12931-023-02496-1 (PMC10413531; doi:10.1186/s12931-023-02496-1)

**Molecular mechanisms and treatment responses of pulmonary fibrosis in severe
COVID-19**

**ADDITIONAL FILE 1**

**METHODS**

**Sensitivity analysis in tocilizumab-treated patients**

To exclude that treatment with tocilizumab in the subgroup of the dexamethasone *(DEXA*)*-cohort* could possibly affect our results, we performed a sensitivity analysis by comparing the number of patients treated with tocilizumab between the pulmonary fibrosis (PF) and no-PF groups of the *DEXA*-*cohort*. Also, the *DEXA-cohort* was subdivided into patients who were treated with tocilizumab and patients who were not and differences in clinical outcomes between the PF and no-PF groups were analyzed within both subgroups (treated with tocilizumab/not treated with tocilizumab).

**Data collection**

Patient characteristics, laboratory data and daily clinical measurements including dynamic lung compliance, PaO_2_/FiO_2_ ratio, minute ventilation, and PaCO_2_ were collected from the electronic patient files (EPF, Epic, Epic Systems Corporation, Verona, Wisconsin, USA) during stay in ICU with a maximum follow up of 40 days. Clinical outcome data (time on ventilator, LOS in ICU and hospital mortality) were also collected from the EPFs. All clinical data were recorded in the Good Clinical Practice (GCP)-certified data management system Castor (Castor EDC, Amsterdam, The Netherlands). Data of minute ventilation and PaCO_2_ were used to calculate daily ventilatory ratio: [minute ventilation (ml/min) × P_a_CO_2_ (mm Hg)]/(predicted body weight × 100 × 37.5).^1^

**Biomarker measurements**

Circulating PIIINP levels were measured in serum and stored at -20⁰C until analysis, three times per week as part of routine care as instructed by the manufacturer using the PIIINP radio immune assay (UniQ®, cat no. 68570, Aidian Oy, Espoo, Finland) and collected from the electronic patient files (EPF). Circulating HGF and MIP-3α levels were measured afterwards by enzyme-linked immunosorbent assays (ELISA) in residual serum samples used for clinical purposes which were stored at -30 ^o^C according to the manufacturer’s instructions (R&D systems Duosets, Minneapolis, Minnesota, USA).

**RNA sequencing**

Of 52 patients of the *pre-DEXA-cohort*, whole blood samples for RNA-seq analysis were collected in PAXgene tubes three times a week following ICU admission. Total RNA was converted into double-stranded cDNA libraries using the TruSeq Stranded Total RNA with Ribo-Zero Globin kit (Illumina). In brief, ribosomal and globin mRNA were depleted from 750 ng purified total RNA using biotinylated, target-specific oligos combined with Ribo-Zero rRNA removal beads; remaining RNA was fragmented using divalent cations under elevated temperature. First-strand was generated using SuperScript2 RT (Invitrogen) supplemented with actinomycin D, followed by second-strand synthesis with dUTP replacing dTTP. 3′ ends were adenylated and index adapters were ligated before subsequent PCR amplification to yield the final library. Remaining overhangs were converted into blunt ends via exonuclease/polymerase activities, and enzymes were removed. Selective enrichment of DNA fragments with ligated adaptor molecules was performed using Illumina PCR primers in a 15-cycle PCR reaction, followed by purification cDNA using SPRIBeads (Beckman Coulter). Libraries were quantified by Qubit dsDNA HS Assay (Thermo Fisher Scientific), and fragment size distribution was determined using the HS D1000 assay on a Tapestation 4200 system (Agilent). High-throughput sequencing was carried out with a NovaSeq™ 6000 Sequencing System S2 (50bp paired-end reads), and data was converted into fastq files using bcl2fastq2 v2.20. The data are made available at the European Genome-Phenome Archive (EGA) under accession numbers EGAS00001005735 and EGAS00001006407, which is hosted by the EBI and the CRG.

**Sequencing alignment and pre-processing**

Sequenced reads were aligned and quantified using STAR: ultrafast universal RNA-seq aligner (v2.7.3a) ^2^ and the human reference genome, GRCh38p13, from the Genome Reference Consortium. For PF-patients, the day prednisone was started was used as alignment day (day 0). For no-PF-patients, the median alignment day of the PF-patients was designated day 0. Raw counts were imported using DESeqDataSetFromHTSeqCount function from DESeq2 (v1.34.0) ^3^. The data was split into two data sets (pre-alignment day: day -12 to day 0; post-alignment day: day 0 to day 6). Both data sets were preprocessed identically. Genes with a lower count than the number of samples were excluded from the analysis. DESeq2 was used for the calculation of normalized counts for protein-coding genes and further relevant gene types using default parameters.

**Gene co-expression network analysis and longitudinal fibrosis-related gene identification**

The gene co-expression network analysis was performed using the hcocena package. Estimation of the number of top variable genes was performed by identifying the inflection point of a curve of the logged variance of the ranked genes. Pearson’s correlation coefficient cut-offs of 0.656 and 0.714 were chosen for the prior to and post-alignment day network, respectively. Gene modules were calculated based on the group fold change (GFC) with the Leiden algorithm iterating it ten times. Database enrichment utilizing the Bioconductor R package clusterProfiler (v4.2.2) ^4^ was performed using the default parameters for the KEGG ^5^ enrichment and HALLMARK ^6,7^ enrichment from the Molecular Signature Database (MSigDB) and using the biological process ontology for the Gene Ontology (GO) ^8,9^ enrichment. Longitudinal genes strongly altered due to fibrosis onset were identified by comparing two linear mixed-effect models (LMEM) for each gene computed using the R package lme4 (v1.1-28) ^10^, of which one includes the fibrosis categorization, over the respective time span using an asymptotic likelihood ratio test from the R package lmtest (v0.9-40) ^11^. Genes with a likelihood ratio Χ²-statistics < 0.1 were defined as statistically significant. Effect size was calculated per gene by squaring the correlation coefficient of the fitted and observed values.

**Decision rules for which CoCena modules to focus on**

For the analysis up to day 0 (pre-alignment day analysis), we focused on modules for which the wave plots showed no intersection of the PF- and no-PF-groups at any timepoint or which showed significant differences (non-overlapping confidence intervals) at day -12 and converged afterwards. For the analysis from day 0 onwards (post-alignment day analysis), we focused on modules for which the PF- and no-PF-groups were significantly different on day 0 and converged afterwards (indicating normalization of the PF-group towards the no-PF-group).

**Survival analysis**

A survival analysis was conducted with the prior to alignment day data set using a microarray data set of idiopathic pulmonary fibrosis (GSE28042, replication cohort) ^12^ as reference data. Gene set variation analysis (GSVA, v1.42.0) ^13^ with the z-score method was used to check the enrichment of the modules filtered for the significant LMEM genes per patient of the reference dataset. Modules with a z-score > 1.645 (p-value ~ 0.1) were specified to be significantly enriched in a patient. Utilizing the survival package (v3.3-1) ^14^ the probability of survival of patients with or without significant enrichment of the gene set were computed per module. P-values were calculated with a log-rank-test and the Kaplan-Meier curves visualized using the survminer package (v0.4.9) ^15^.

**Statistical analysis**

To analyze kinetics of serially measured data, variables were binned into bins spanning two or three days because not all variables were measured daily in all patients. Because of the relatively small size of the cohorts, a normal distribution of data was not assumed. Data are displayed as median with interquartile ranges [IQR], numbers with percentages, or geometric means with 95% confidence intervals (CI). P-values <0.05 were considered to indicate statistical significance. Differences in baseline characteristics and clinical outcomes between the PF- and no-PF-groups were analyzed using Mann-Whitney U and Fisher’s exact tests for continuous and categorical data, respectively. Differences in kinetics of serially measured data were analyzed using linear mixed effect model analysis on log-transformed data followed by post-hoc Sidak’s multiple comparisons tests. Because data collection was only performed during stay in ICU, we performed Last Observation Carried Forward (LOCF) for data of patients who were discharged from ICU or who deceased. LOS in ICU and mortality were analyzed using log-rank tests during 60 days following ICU admission. For the log-rank test of 60-day mortality, patients who were discharged alive from the hospital or were still in the ICU or hospital on day 60 were censored at day 60. Statistical analysis was performed using SPSS 25 (IBM SPSS statistics version 25.0. Armonk, NY: IBM Corp) and GraphPad Prism 8 (GraphPad Software, La Jolla California, USA).

**REFERENCES**

1. Sinha P, Calfee CS, Beitler JR, et al. Physiologic Analysis and Clinical Performance of the Ventilatory Ratio in Acute Respiratory Distress Syndrome. *American Journal of Respiratory and Critical Care Medicine.* 2018;199(3):333-341.

2. Dobin A, Davis CA, Schlesinger F, et al. STAR: ultrafast universal RNA-seq aligner. *Bioinformatics.* 2013;29(1):15-21.

3. Love MI, Huber W, Anders S. Moderated estimation of fold change and dispersion for RNA-seq data with DESeq2. *Genome Biology.* 2014;15(12):550.

4. Wu T, Hu E, Xu S, et al. clusterProfiler 4.0: A universal enrichment tool for interpreting omics data. *Innovation (Camb).* 2021;2(3):100141.

5. Kanehisa M, Furumichi M, Tanabe M, Sato Y, Morishima K. KEGG: new perspectives on genomes, pathways, diseases and drugs. *Nucleic Acids Research.* 2017;45(D1):D353-D361.

6. Subramanian A, Tamayo P, Mootha Vamsi K, et al. Gene set enrichment analysis: A knowledge-based approach for interpreting genome-wide expression profiles. *Proceedings of the National Academy of Sciences.* 2005;102(43):15545-15550.

7. Liberzon A, Birger C, Thorvaldsdóttir H, Ghandi M, Mesirov Jill P, Tamayo P. The Molecular Signatures Database Hallmark Gene Set Collection. *Cell Systems.* 2015;1(6):417-425.

8. Ashburner M, Ball CA, Blake JA, et al. Gene Ontology: tool for the unification of biology. *Nature Genetics.* 2000;25(1):25-29.

9. The Gene Ontology C. The Gene Ontology resource: enriching a GOld mine. *Nucleic Acids Research.* 2021;49(D1):D325-D334.

10. Bates D, Mächler M, Bolker B, Walker S. Fitting Linear Mixed-Effects Models Using lme4. *Journal of Statistical Software.* 2015;67(1):1 - 48.

11. Zeileis A, Hothorn T. Diagnostic Checking in Regression Relationships. 2001;2.

12. Herazo-Maya Jose D, Noth I, Duncan Steven R, et al. Peripheral Blood Mononuclear Cell Gene Expression Profiles Predict Poor Outcome in Idiopathic Pulmonary Fibrosis. *Science Translational Medicine.* 2013;5(205):205ra136-205ra136.

13. Hänzelmann S, Castelo R, Guinney J. GSVA: gene set variation analysis for microarray and RNA-Seq data. *BMC Bioinformatics.* 2013;14(1):7.

14. Therneau TM, Grambsch PM. Modeling Survival Data: Extending the Cox Model *Springer.* 2000.

15. Kassambara A, Kosinski M, Biecek P, Scheipl F. Drawing Survival Curves using 'ggplot2'. 2021.

**TABLES**

**Table S1.** Clinical outcomes within tocilizumab subgroups of the *DEXA-cohort*.

Data presented as n (%) median with interquartile ranges ([IQR]). P-values were calculated using Mann-Whitney U and two-sided Fisher’s exact tests for continuous and categorical data, respectively.

DEXA: dexamethasone, PF: pulmonary fibrosis, ICU: intensive care unit

|  | Not treated with tocilizumab | | | Treated with tocilizumab | | | PF + no tocilizumab vs. PF + tocilizumab |
| --- | --- | --- | --- | --- | --- | --- | --- |
|  | PF (n=11) | No-PF (n=37) | p-value | PF (n=20) | No-PF (n=56) | p-value | p-value |
| Hospital mortality | 7 (64) | 9 (24) | 0.02 | 12 (60) | 9 (16) | <0.001 | 1.00 |
| Length of stay in ICU (days) | 42 [22-70] | 12 [8-25] | <0.001 | 41 [28-50] | 15 [8-24] | <0.001 | 0.68 |
| Survivors | 56 [27-73] | 11 [8-22] | 0.005 | 47 [36-66] | 16 [8-23] | <0.001 | 1.00 |
| Non-survivors | 29 [21-64] | 18 [12-39] | 0.02 | 32 [22-47] | 12 [7-25] | 0.007 | 0.61 |
| Time on ventilator (days) | 34 [20-61] | 8 [5-20] | <0.001 | 30 [21-49] | 12 [3-20] | <0.001 | 0.80 |
| Survivors | 49 [24-62] | 7 [4-18] | 0.005 | 40 [23-61] | 11 [4-18] | 0.001 | 0.87 |
| Non-survivors | 28 [20-59] | 13 [8-36] | 0.04 | 30 [21-44] | 14 [0-22] | 0.004 | 1.00 |

**FIGURES**

**Figure S1.** Complete analysis of bulk RNA-seq data pre-alignment day (day 0, the day prednisone treatment was initiated in patients with PF). **(a)** displays the mean expression per module filtered by the LMEM genes over time prior to the alignment day. Lines and confidence intervals are colored according to the condition. **(b)** depicts the mean expression of the top 10 LMEM genes per module ordered by effect size for all conditions and time points prior to the alignment day. Modules are colored accordingly and effect size is indicated by the dot size. **(c)** shows the top 3 significant functional enrichment terms from GO and KEGG database as well as the hallmark gene set of the Molecular Signature Database per module. Modules names are displayed on the x-axis and the respectively colored squares indicate the enrichment of a functional term in the module.


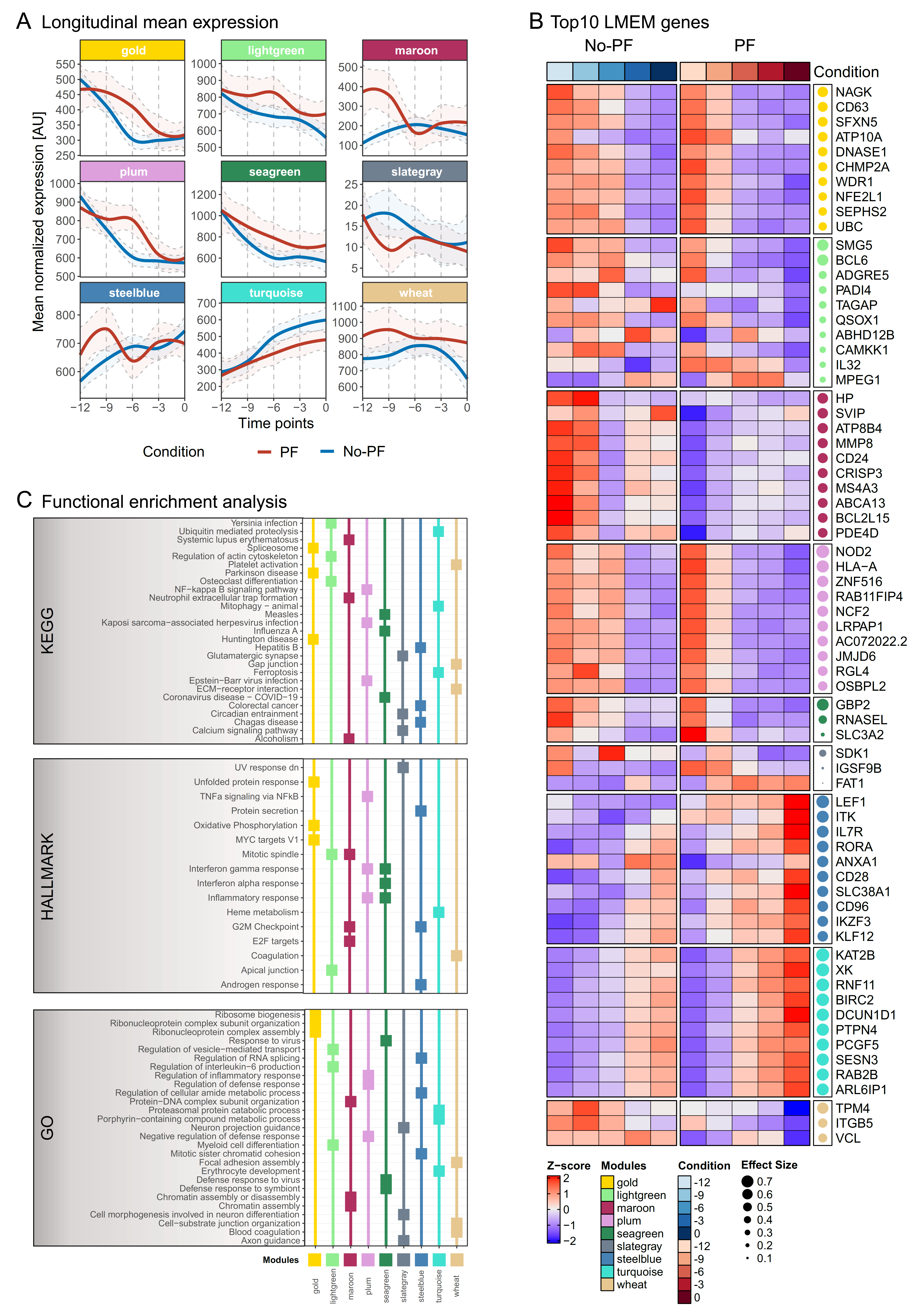


**Figure S2.** Survival analysis of bulk RNA-seq data pre-alignment day (day 0, the day prednisone treatment was initiated in patients with PF).

**(a)** Survival curves of all modules. Lines are colored based on the enrichment of the LMEM genes in the respective module in the reference dataset using GSVA.


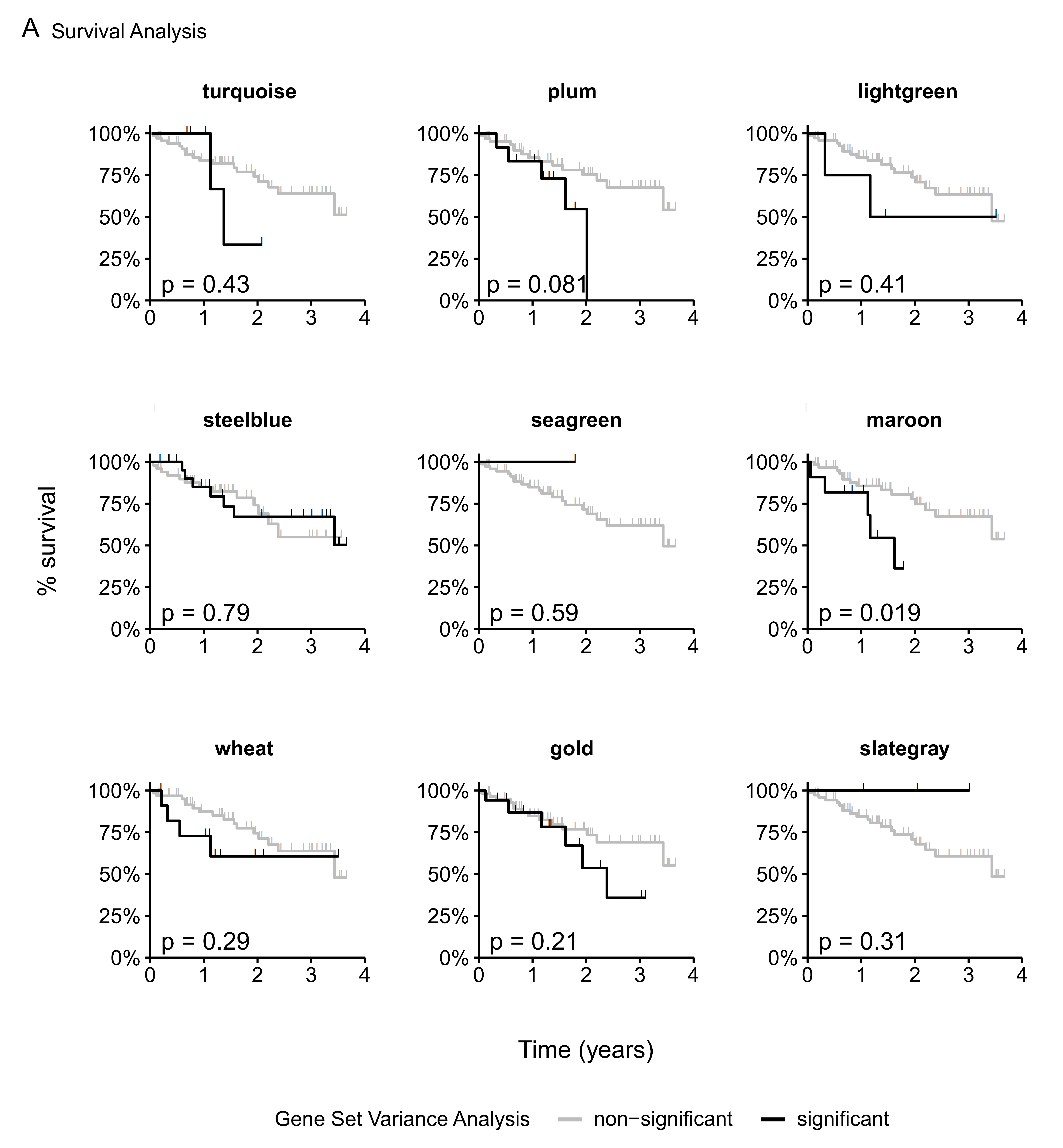


**Figure S3.** Complete analysis of bulk RNA-seq data post-alignment day (day 0, the day prednisone treatment was initiated in patients with PF). **(a)** displays the mean expression per module filtered by the LMEM genes over time post-alignment day. Lines and confidence intervals are colored according to the condition. **(b)** depicts the mean expression of the top 10 LMEM genes per module ordered by effect size for all conditions and time points post-alignment day. Modules are colored accordingly and effect size is indicated by the dot size. **(c)** shows the top 3 significant functional enrichment terms from GO and KEGG database as well as the hallmark gene set of the Molecular Signature Database per module. Modules names are displayed on the x-axis and the respectively colored squares indicate the enrichment of a functional term in the module.


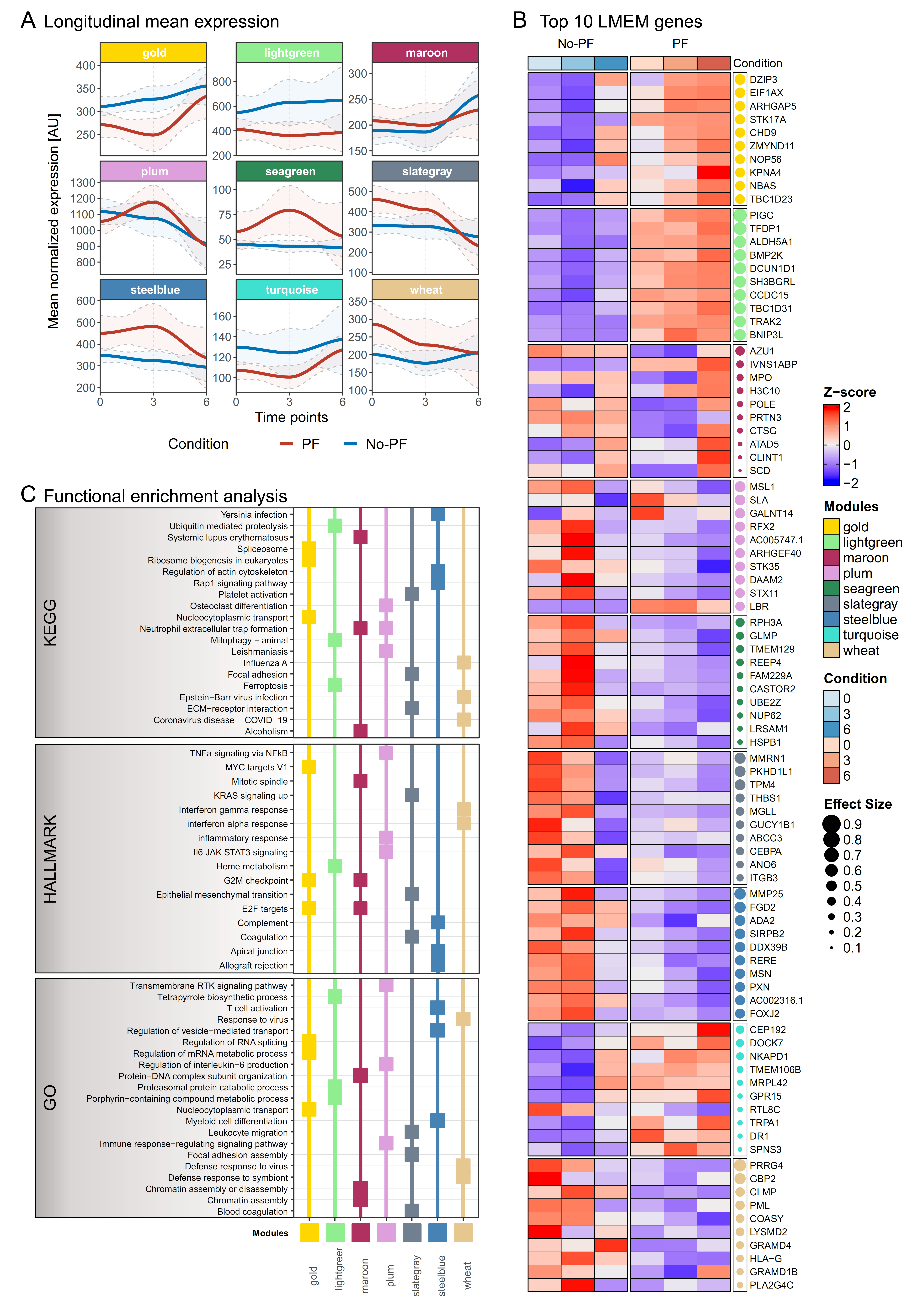


**Figure S4.** Circulating fibrosis markers.

Differences between the pulmonary fibrosis (PF)- and no-PF groups in kinetics of circulating hepatocyte growth factor (HGF) in **(a)** *pre-DEXA-cohort* and **(b)** *DEXA-cohort* and

Macrophage Inflammatory Protein 3 Alpha (MIP-3α) in **(c)** *pre-DEXA-cohort* and **(d)** *DEXA-cohort* within 6 days prior to and following the alignment day (PF-day 0, start of prednisone treatment in the PF-groups). P-values on the left and the right of each panel reflect between-group differences over time for the days prior to and following PF-day 0, respectively, and were calculated using linear mixed models analysis (time*group interaction factor). Data presented as geometric mean with 95% confidence intervals. * p-value <0.05 on the corresponding timepoint, calculated using Sidak’s post-hoc multiple comparisons test.





**Figure S5.** **Ventilatory parameters of the pulmonary fibrosis groups.**

Differences between the pulmonary fibrosis (PF)-groups of both the *pre-DEXA-* and *DEXA-cohorts* in kinetics of **(a)** dynamic lung compliance, **(b)** ventilatory ratio, and **(c)** PaO_2_/FiO_2_ ratio within 8 days prior to and 12 days following the alignment day (PF-day 0, start of prednisone treatment in the PF-groups). P-values on the left and the right of each panel reflect between-group differences over time for the days prior to and following PF-day 0, respectively, and were calculated using linear mixed models analysis (time*group interaction factor). Data presented as geometric mean with 95% confidence intervals.


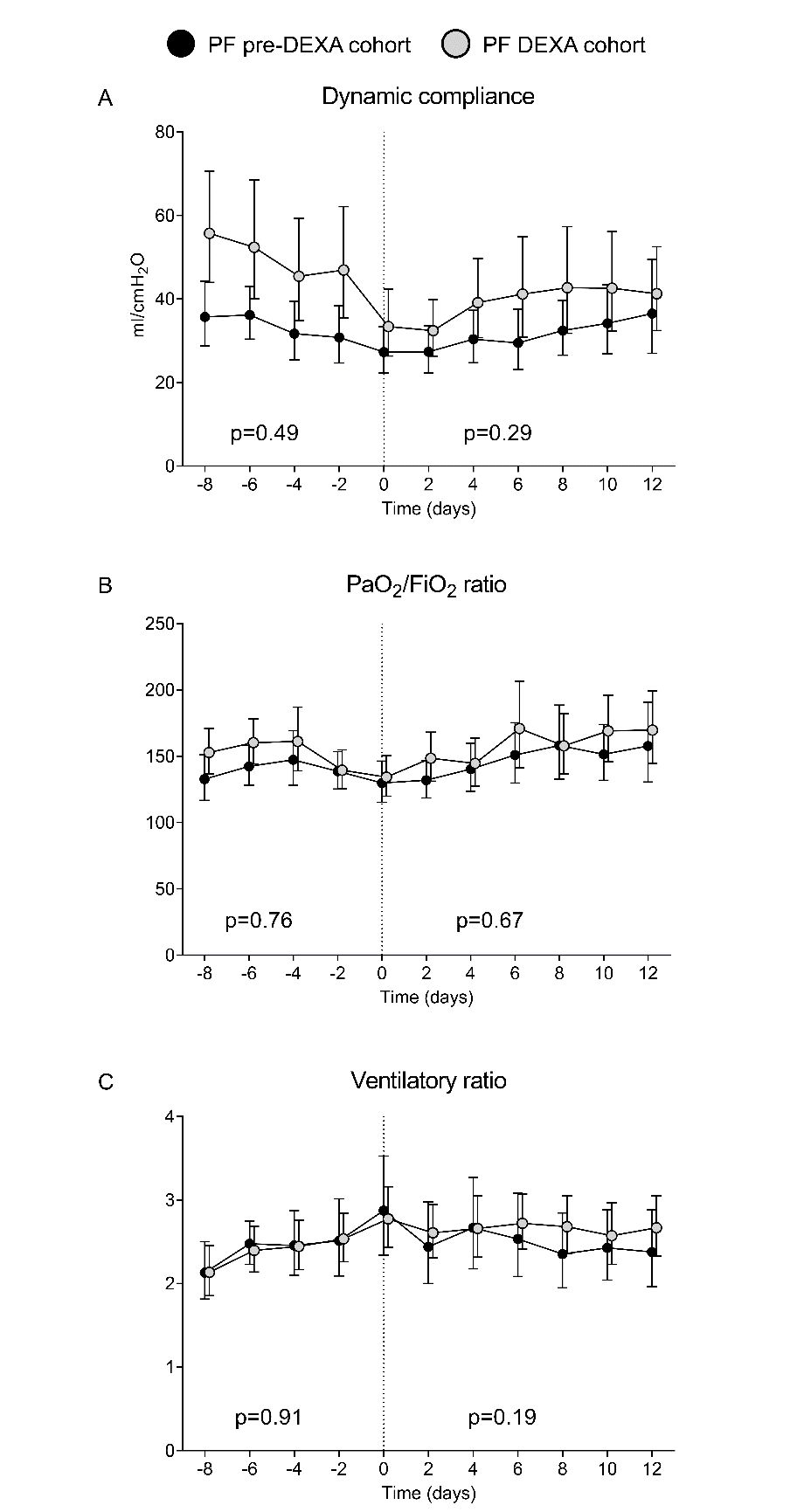

Supplement: Supplementary file 1 — Additional file 1. Additional Methods, Additional Table S1 and Additional Figures S1–S5. [file 12931_2023_2496_MOESM1_ESM.docx]
